# Physiological Profile, Metabolic Response and Temporal Structure in Elite Individual Table Tennis: Differences According to Gender

**DOI:** 10.3390/ijerph182211898

**Published:** 2021-11-12

**Authors:** Francisco Pradas, Ana de la Torre, Carlos Castellar, Víctor Toro-Román

**Affiliations:** 1 ENFYRED Research Group, Faculty of Health and Sports Sciences, University of Zaragoza, 22001 Huesca, Spain; franprad@unizar.es (F.P.); 678853@unizar.es (A.d.l.T.); castella@unizar.es (C.C.); 2 School of Sport Sciences, University of Extremadura, 10003 Cáceres, Spain

**Keywords:** game density, cardiac response, oxygen consumption, lactate, racket sport

## Abstract

No research that has analyzed the structural characteristics, physiological profile, and energy demands in the game of table tennis as played by women is available. The present study aimed to evaluate the physiological, metabolic, and temporal variables of table tennis players and to observe gender differences. Forty-eight elite table tennis players participated in this study: 24 men (25.3 ± 4.07 years) and 24 women (22.3 ± 3.8 years). During simulated competition, temporal structure, heart rate (HR), and lactate (LA) were evaluated. The maximum ergospirometric evaluations were performed in a laboratory. The total table tennis (TT) time and the total resting time (TRT) were longer for men (*p* < 0.05), but game density was higher for women (*p* < 0.05). During rallies, the real playing time (RPT) was longer for women, while the TRT was longer for men (*p* < 0.05). The maximum HR, minimum HR, and maximum LA concentrations were higher for men (*p* < 0.05). The obtained data reveal gender differences in the physiological, metabolic, structural, and temporal variables in table tennis players. The analysis of the studied variables could allow training sessions to be planned and organized according to table tennis players’ gender.

## 1. Introduction

Table tennis is one of the most well-known and widely played racket sports in the world with more than 300 million who practice this sport, of whom at least 40 million are federated players [1,2]. In the last two decades, this sport has considerably changed with relevant modifications made to its regulations and game characteristics, such as including plastic balls to replace celluloid ones, increased ball diameter and weight, a concise points system that has gone from 21 to 11 points, time-outs being introduced, harmful substances used to stick rackets’ rubber coatings being forbidden, or even a key basic technical aspect in this sport like its service being reformed [3]. These modifications have brought about changes in various fundamental table tennis aspects, and considerably differ from those disputed some years ago insofar as physiological and metabolic demands are concerned, in the game’s structure, or even physical demands [4]. Hence, this game may evolve differently when played by one gender or another.

Yet despite table tennis being extremely popular, its long-standing history and major modifications to its regulations and game, very few scientific studies have investigated this sport and both genders. Research on this topic is very heterogeneous and decontextualized, and does not contemplate the female gender. On the one hand, lack of studies is a direct consequence of the sport’s complex nature because it is not easy for scientists to provide measures and to collect the necessary data to present them to trainers and players [4]. On the other hand, to this day, scientific interest in addressing this sport in general, and women’s table tennis in particular, has been scarce.

In order to more accurately know the physiological and metabolic demands involved in playing table tennis, it is necessary to learn the game’s dynamics. Thus, describing game parameters like temporal structure by considering this sport’s playing times and total resting times (TRT), as well as games’ effort times and resting times, are very interesting study variables and also determining factors to know the organic impact of playing table tennis, as well as the possible differences between men and women’s table tennis games [5].

The research works conducted to deal with total match duration have examined the duration of masculine competition, and found that it ranges from 8 to 38 min, and female competition between 9 and 41 min [5,6,7,8], and some of the world’s top players can even play for a maximum time of 45 min [3]. The average rally time ranges between 3.1 and 4.6 s in men [9], but no data were found about this temporal game variable for women.

In physiological terms, heart rate (HR) and its different manifestations—such as maximum (HR_max_), mean (HR_mean_), and minimum (HR_min_)—are generally studied parameters for forming one of the few direct physiological indices applied during competition without altering its essence. Those studies that have analyzed HR_mean_ to determine the intensity of table tennis obtained mean values of 135–163 beats·min^−1^ [7] or 136–147 beats·min^−1^ [10]. Moreover, HR_max_ values have been reported to fall within the ranges of 177–183 beats·min^−1^ [7,8] or 159–173 beats·min^−1^ [10]. Therefore, efforts are made in table tennis with wide cardiac variability where submaximum-type efforts predominate, but at a high HR (from 68% to 92% of HR_máx_) [8]. As an indirect method, %HR_max_ is another option to determine the percentage of cardiovascular effort made while playing table tennis.

Maximal oxygen uptake (VO_2_) is employed in its maximal aerobic capacity expression (VO_2max_) and its use as a percentage (%VO_2max_), and is another valid indicator to know the effort. Kondrič et al. [11] indicate that VO_2max_ is one of the most widely used physiological parameters that researchers apply to determine the energy demands needed to play table tennis. Table tennis players’ maximum physiological responses are normally analyzed in laboratories by testing [7,10,12,13]. These studies reveal that top table tennis players present VO_2max_ values of 41–63.1 mL·kg·min^−1^ [7], 42.8–48.2 mL·kg·min^−1^ [10] or 40.3–45.7 mL·kg·min^−1^ [12]. These values indicate the intervention and the importance as a percentage of the aerobic system in this sport.

Knowledge and evaluations of the involved metabolism are another extremely important matter to know the physiological and physical demands of table tennis. Zagatto et al. [12] offer information about the contribution of each metabolic profile while playing a table tennis match. These researchers found that 96.5% of energy came from oxidative metabolism, 2.5% from phosphagen metabolism (ATP-PCr), and 1.0% from glucose metabolism. Oxidative and glucose energy demands are closely related to rally duration. Moreover, blood lactate (LA) concentration can be taken as a suitable marker to establish the degree at which the different energy systems involved in table tennis are demanded. Recent research studies performed in top players indicate that the LA response in table tennis is between 1.6 and 2.9 mmol·L^−1^, with averages of 1.7 mmol·L^−1^, and maximum peaks of 2.1 mmol·L^−1^ [7,8,14].

Most of the research works that have analyzed this sport’s structural characteristics, and its physiological profile and energy demands, have done so only in men, and these topics studied in women are practically nonexistent. Likewise, studies that have investigated this matter have been performed with only a few players [12,14]. Gender differences in reaction time and lateral movements have been previously observed [15]. We believe that these differences may influence the temporal structure, physiology, and metabolic response of the players. For these reasons, the objective of the present study was to evaluate the most relevant physiological, metabolic, and temporal variables associated with individual top table tennis players’ performance according to gender.

## 2. Materials and Methods

### 2.1. Participants

Forty-eight top table tennis players voluntarily participated in this study: 24 men (25.3 ± 4.07 years) and 24 women (22.3 ± 3.8 years). These table tennis players were recruited from sport clubs and had to meet the following criteria: (i) be older than 18 years old; (ii) participate in the top category of the Spanish league; (iii) play in international competitions; and (iv) not have any injuries or illness during the investigation or at least 6 months before the study. After receiving both comprehensive verbal and written explanations of the study, written informed consent was obtained from parents or legal tutors. The Ethics Committee of the University of Zaragoza (ID:19/2010) reviewed and approved the study. All athletes participated voluntarily in the research, were informed about the aim of the study, and gave their written informed consent. A code was assigned to each participant to collect and process data to maintain their anonymity. The specific table tennis training volume and the complementary training volume (strength, mobility, and flexibility) were determined.

### 2.2. Anthropometric Evaluation

Body mass (kg) and height (m) were collected using a scale (Seca 769, Seca, Hamburg, Germany) and a measuring rod (Seca 220, Seca, Hamburg, Germany) with an accuracy of ±0.001 kg and 0.001 m under nude barefoot conditions. Body mass index (BMI) was calculated from the body mass and stature ratio.

### 2.3. Laboratory Measurements

A maximum progressive test was run in the laboratory on a treadmill (Pulsar HP, Cosmos, Nussdorf, Germany) to determine physiological parameters HR_max_ and VO_2max_ and the metabolic performance values of LA. This test was performed at a 1% slope starting at a speed of 8 km·h^−1^ and incorporating 1 km·h^−1^ increments every min. Before testing began, the participants warmed up on a treadmill at the speed of 7 km·h^−1^ for 7 min. Gases were analyzed by an Oxycon Pro analyzer (Jaegger, Germany). A pulsometer (Polar Team System, Kempele, Finland) was used to evaluate HR_max_. The LA concentration was evaluated when testing ended by a photoenzymatic analysis (Dr. Lange LP-20, Berlin, Germany) after the same researcher had taken 20 µL capillary blood samples from ear lobes following the guidelines of Feliu et al. [16].

### 2.4. Competition

Given the difficulties of analyzing acute metabolic and physiological parameters during table tennis matches, a simulated competition (SC) was designed. The SC consisted of organizing a table tennis match for reproducing a competition like official ones and in accordance with International Table Tennis Federation (ITTF) rules. All matches were played to the best of seven sets. Before each match, players did a standardized 15-min warm-up divided into a 5-min movement and a general warm-up session and a 10-min specific technical warm-up on the court. Total time (TT; full match time, from the beginning to the end, considering game and rest periods), TRT (sum of the periods during which the ball was not played), and real playing time (RPT; total time, less the TRT) were measured (s) during each match. Apart from these variables, game density (playing/resting times) was also analyzed. Moreover, players’ HR was continuously recorded during the competition (Polar Team System, Kempele, Finland) as average values over 5 s.

During the SC, blood samples were taken to evaluate the LA concentration before a match started, when each set finished and at minutes 1, 3, and 5 after matches ended. Table tennis matches were held with environmental relative humidity and a temperature of 48 ± 2.6% and 22 ± 0.8 °C, respectively.

### 2.5. Temporal Structure

**Figure 1:**
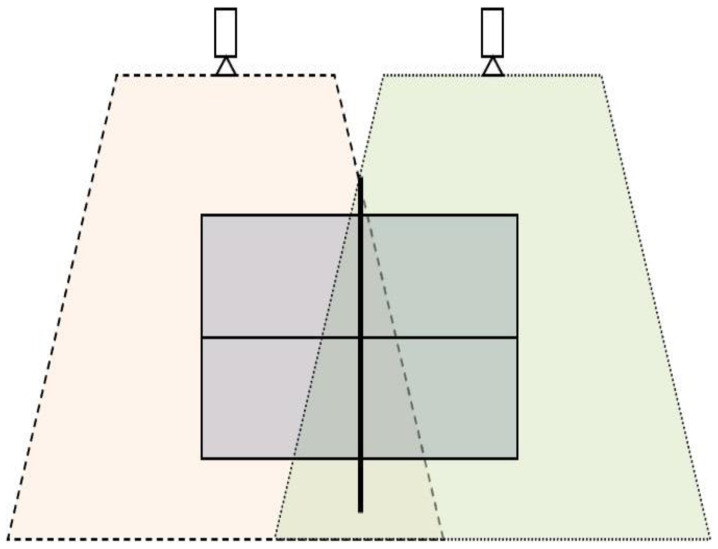
Video-recording protocol.

SCs were recorded using Sony HDR-CX300E video cameras (Sony, Tokio, Japan), which were set up on telescopic supports (Manfrotto 007U, Cassola, Italy) on the sides of table tennis tables at a minimum distance of 3 m and a height of 2.5 m. Matches were recorded at a shutter speed of 1/500 s. Each video camera recorded half a table (Figure 1), and two recordings were obtained, which included how each player played.

Following these recordings, both cameras underwent a synchronization process in the laboratory. Agreements were analyzed by an observation tool validated for table tennis [17] using Match Vision Studio©, v. 3.0, which was run by a notational ad hoc system that allowed effort times and pause times to be studied. Two observers with physical activity and sports degrees, who were qualified expert trainers in top-level table tennis, analyzed the table tennis matches. The temporal data-agreement analysis gave a Kappa Index value above 0.80 for all the studied variables, which is considered to be a very high level of agreement [18].

### 2.6. Statistical Analysis

Means, standard deviations (SD), and range (min–max) were described. The statistical analysis was run with Version 22 of IBM^®^ SPSS^®^ Statistics (IBM Corp., Armonk, NY, USA). The Shapiro–Wilks test was used to determine the normal distribution of the variables. Homogeneity of variances was examined by the Levene test. A Student’s *t*-test was performed with the unrelated samples to observe any gender differences. A two-way analysis of variances (ANOVA) was applied to analyze any differences in the LA values that took place during the SC (gender x time). A *p*-value of ≤ 0.05 was considered to be statistically significant.

## 3. Results

Table 1 shows players’ maximum values obtained in maximal incremental test performed in the laboratory. In the studied variables gender differences were found for all the variables, except HR_max_ (*p* < 0.05).

Table 2 show the game structure analysis results. It is highlighted in the male category that 16.67% of the played matches included five sets, 66.17% had six sets and 16.67% included seven sets. For the female category, 58.33% of the matches included five sets, 33.33% had six sets and 8.33% included seven sets. The temporal structure analysis indicated that both TT and TRT were significantly longer in the male competition than in the female one (*p* < 0.05). Nevertheless, women’s game density was higher (*p* < 0.01). Significant gender differences appeared for both RPT and TRT for rallies (*p* < 0.05).

**Figure 2:**
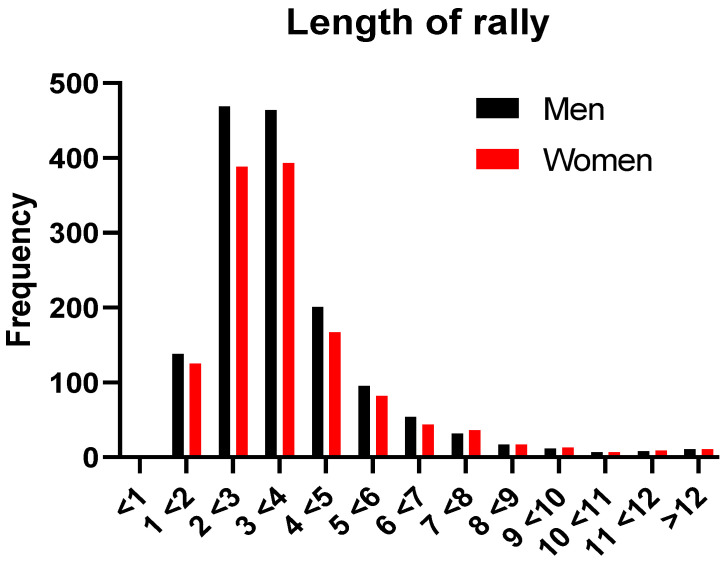
Rally duration distribution per gender.

Figure 2 illustrates the rally duration frequency for both sexes, where we can see that frequencies are higher for the rally durations between 2 and 4 s for both sexes.

Table 3 presents the results corresponding to the analysis of the physiological and metabolic responses during the SC. All the HR-related variables were higher in the male competition. The mean and the maximum HR cardiac responses were similar for both men and women.

**Figure 3:**
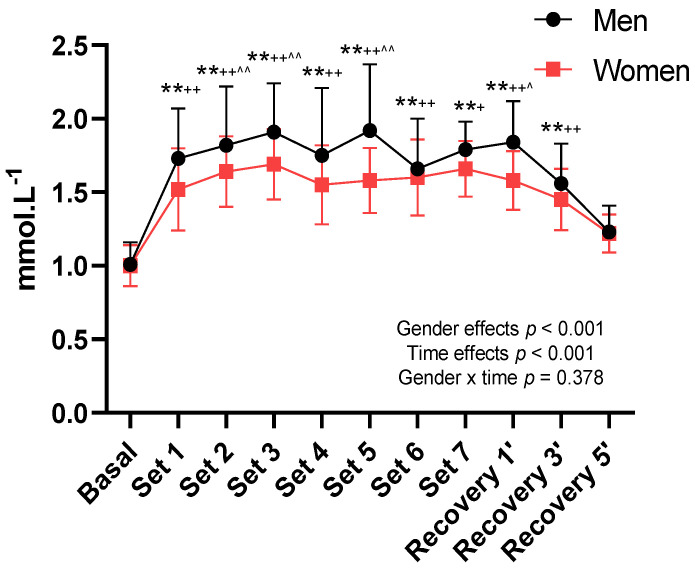
LA evolution during SC in men and women; ** *p* < 0.01 Basal vs. other measurements; + *p* < 0.05 Recovery 5′ vs. other measurements; ++ *p* < 0.01 Recovery 5′ vs. other measurements; ^ *p* < 0.05 Recovery 3′ vs. other measurements; ^^ *p* < 0.01 Recovery 3′ vs. other measurements.

In metabolic terms, the maximum LA levels were significantly higher in the male competition (*p* < 0.001), with maximum LA peaks during matches of 2.5 mmol·L^−1^ for male players and 1.9 mmol·L^−1^ for female players. Significant gender differences were observed in LA evolution throughout both the SC (Figure 3) (*p* < 0.001).

## 4. Discussion

This study aimed to evaluate the physiological, metabolic, and temporal variables associated with sport performance in individual top tennis table players according to gender. As far as we know, this is one of the first research works to systematically analyze the impact of modern table tennis on female players. Due to the differences in age and experience between the two groups (Table 1), we decided to conduct an additional statistical analysis to check the influence of these variables on the results. However, after conducting the analysis, these variables did not significantly affect the results.

In order to accurately know the physiological and metabolic demands that playing table tennis involves, it is necessary to previously know its game dynamics. Therefore, describing game parameters like temporal structure is very interesting and a determining factor to know both the impact of playing table tennis and any possible existing gender differences.

Research that has studied TPT in regional, national, and Olympic competitions reports how masculine competition times last between 8 and 38 min, and last from 9 to 41 min for women [5,6,7,8]. Authors like Kasai et al. [19] reported that Japanese men’s TPT is around 30 min, which can even last up to 45 min with top world players [3]. The results obtained in the present study are in agree with the reviewed studies that respectively place feminine competition and male competition in approximately 24 min and 37 min.

Thus, the temporal rally duration range is large. These variations in the temporal structure of matches can be due to the different parameters that impact the temporal variable, such as a (local, regional, national, or international) competition’s level of demand, competition category (played to the best of five or seven games), the competition phase players are in (preliminary rounds, rounds of 16, quarterfinals, semifinals, final), the studied players’ level (amateurs, university students, professionals), and even the more or less defensive game type that predominates in competitions [3,8,13].

Another important variable to analyze the temporal structure, and one that allows the physical demand related to the efforts made when playing table tennis to be quantified, is rally duration. Katsikadelis et al. [6] found that the playing times per game in the 2004 Athens Olympic Games were 4.18 ± 0.75 s for men and 5.04 ± 0.81 s for women. Similar results were obtained for the 2008 Beijing Olympic Games, where rally durations were 5.0–7.3 s in the female competition and 4.5–5.3 s in the male competition [20]. The specialized literature indicates some results showing longer rally duration in female competition, as the present work does, but the results are lower rally duration was 4.03 ± 1.0 s for women and 3.6 ± 0.3 s for men [7,21]. These differences can be explained by the studied players’ high level, the style played given a higher diversity of mixed and defensive games, and also by the studied competition type, namely Olympic and World table tennis matches, considered to be the best ones internationally and the competitions in which the world’s top players participate.

After analyzing the data obtained in the games, the temporal records obtained in the present study indicate a higher effort density in individual women’s matches (0.41 ± 0.1) than for men (0.27 ± 0.05). The results analyzed in this research fall in line with those described for male table tennis [3,8,12]. Nevertheless, it is worth highlighting once more that game density can also be affected by players’ game level, game style, and the competition level/phase, which are extremely variable densities ranging from 0.12 to 0.5 [9]. No research works describing the game densities of female table tennis are available.

When comparing the temporal structure of table tennis to other racket or bat sports, game times are longer in badminton with values of 6.8 s in male competition and 4.3 s in female competition [22]. In tennis, points duration are longer than in table tennis, with game times of 5.2 s for men and 7.1 s for women [23]. Finally in padel, duration is also longer than table tennis with game effort values between 9.3–11.7 s in men and 9.6–13.03 s in women [7,24].

The table tennis game density encountered in this research is lower than that found for other racket and bat sports like tennis [25] or badminton, where values of 0.53–0.57 have been described for male competition and 0.47 for female competition [26,27].

Obtaining data in table tennis from a physiological point of view without influencing competitions is complicated. In the scientific literature, the most widely analyzed physiological and metabolic parameters in this group tend to be ventilation-type parameters like VO_2max_ [6,19,28], cardiac response [8,12,13,19], and blood LA concentration [10,14,28].

VO_2max_ is considered an ideal marker for knowing a player’s physical aerobic condition [11]. Some studies performed with top Asian and European players have revealed VO_2max_ levels of 43.9–67.9 mL·kg·min^−1^ in incremental strength testing [13,29,30,31], which are similar to those reported. These values correspond to male players and the specialized literature does not cite any reference values for women. This study obtained VO_2max_ values of 32.6–54.3 mL·kg·min^−1^ for women.

Cardiac response is another relevant and widely investigated parameter in table tennis [8,11,13]. The analysis of this variable is very interesting to understand the physiological demand in the physical demand terms that players are subjected to while playing. The values recorded for the players in the present study are similar to those described by other research works [19,32], and no reference data were found for female table tennis players. Analyzing cardiac response is important for performance because positive relations between players’ levels and response to HR during recovery periods have been reported [30,32].

Research conducted into official competitions and SCs place HR_max_ within the 160–180 beats·min^−1^ range for male players [19,32]. These results agree with this maximum record obtained from male competition, with a maximum peak of 177 beats·min^−1^. No such reference data were found in the specialized literature for female table tennis players. This study observed that the maximum cardiac response in female competition was 139–167 beats·min^−1^, with average records of 155.4 ± 8 beats·min^−1^, and a maximum cardiac response lower than that obtained for male players.

However, HR_max_ could be different [30] depending on the game style played by both gender (offensive, mixed, defensive), and also on the technical-tactical game played [13,19]. Moreover, using certain individual sport materials (wood and rubber coatings) and specific competition situations, such as scores on scoreboards or a decisive point of the played game [33], are psycho-physiological variables that can affect high cardiac reactivity and may substantially impact HR and VO_2_ [34].

Analyzing HR_mean_ is another cardiac variable used to know the intensity. Many research works conducted before the ITTF made changes to regulations centered on describing the HR_mean_ of a match, which they reported as falling within the 137–176 beats·min^−1^ interval in male competitions [13,19,29]. Once again, no reference data about female competition were found in the literature. The present research obtained average cardiac records of 138.8 ± 12.1 beats·min^−1^ for men and 137.2 ± 6.03 beats·min^−1^ for women, which are slightly lower than those in the previously described research works. The differences found in today’s table tennis can, once more, be due to the changes made to sport materials and ball size, which today have a bigger diameter. All this slows down today’s tennis table playing [35,36,37], which could directly influence HR_mean_, as could some other changes made by the ITTF.

Another cardiac parameter applied to evaluate individual efforts while playing competitions is %HR_max_. Allen [29] and Yuza et al. [13] respectively conducted studies with the former regulations on top Australian and Japanese players. They indicated that %HR_max_ in a top-level match was 71–86% of HR_max_. Likewise in matches played in line with current regulations by top-level players with international experience, Suchomel [30] and Zagatto et al. [8] reported HR_max_ values that fell within a maximum range that was slightly narrower than those previously described, with %HR_max_ between 78% and 81.2%. Once more, no data for female competitions were found. The present research results indicate lower strength values than those described for male competition, with approximately 71% of HR_max_ for men and 70% of HR_max_ for women.

Finally, blood LA concentration can be used as a suitable marker to determine the degree of metabolic demand. LA is the final product of aerobic glycolysis that depends on the intensity and duration of effort [8]. In metabolic terms, research conducted on top players indicates that lactic response in table tennis is between 1.6 and 2.9 mmol·L^−1^, with averages of 1.7 mmol·L^−1^ and maximum peaks of 2.1 mmol·L^−1^ [7,8,14]. These results fall within the ranges shown in the present work, where male players achieved an average of 1.8 mmol·L^−1^ and maximum peaks of 2.5 mmol·L^−1^. No publications were found that describe the metabolic impact of playing table tennis on female players. Nonetheless, the values obtained in female competition in the present study were lower than those found for male competition, with average LA values of 1.5 mmol·L^−1^ and maximums of 1.9 mmol·L^−1^. This demonstrates acceptable differences between the games played by men and women.

This sport involves short rally durations and lots of pauses, which could influence LA concentration. Low LA production could result from the ATP-PCr system (the phosphagens system) predominating during effort periods because, as previously mentioned, the rally duration in both genders did not exceed an average of 4.5 s, and also due to oxidative metabolism predominating in rest/pause times. According to our results and the literature, it can be confirmed that anaerobic glycolysis is rarely required to obtain energy while playing table tennis [8].

The analysis of the obtained data showed differences in maximum LA concentrations men and women. These differences could be due to the difference in each gender’s muscle mass [38]. Moreover, it is known that women tend to obtain lower respiratory coefficient values, high fat oxidation rates, and lower blood LA values [39,40].

LA values recorded during matches be interpreted with caution because LA samples are taken after playing different sets. This could entail clearance as a result of game pauses and the time between ending games and taking samples. Another factor to contemplate is the game style played, because it seems to strongly influence metabolic response. Indeed, average LA values of 4.7 mmol·L^−1^ with maximum peaks of 6.1 mmol·L^−1^ have been reported during matches played with an offensive player against a defensive one [41].

The present research is not without its limitations: (i) VO_2max_ was not evaluated during the SC or by any specific test; (ii) players’ game style (defensive, mixed, offensive) was not taken into account; (iii) no possible existing relation was analyzed between the employed sport materials (wood/rubber coatings), which determine the played game style, and the player’s distance from the table (close, medium, far), which is a very important factor in table tennis given its high technical-tactical demands; (iv) the difficulty of taking LA samples during each game because it is practically impossible, so values can fluctuate depending on the game’s dynamics and playing action before taking samples.

## 5. Conclusions

The data obtained reveal gender differences for the physiological, metabolic, structural, and temporal variables in table tennis players.

Male players’ TT and TRT are longer than they are for female players. The RPT for rallies was shorter for male players and TRT was shorter for female players.

Regarding the physiological variables, male players obtained higher HR_max_ and HR_min_ values during matches than female players. The maximum LA values during matches were higher in male players.

The variables studied in this research allowed us to better understand the temporal structure and physiological demands needed to thoroughly plan and organize training according to gender.

## Figures and Tables

**Table 1:** Table tennis players’ characteristics.

Variable	Men		Women		*p*
	M ± SD	Range	M ± SD	Range	
Age (y)	25.3 ± 4.07	19–38	22.3 ± 3.8	18–31	0.007
Height (m)	1.75 ± 0.06	1.62–1.68	1.65 ± 0.06	1.52–1.75	<0.001
Weight (kg)	69.9 ± 9.2	50.8–89.6	57.6 ± 6.2	48.3–69.8	<0.001
BMI (kg·m^−2^)	22.6 ± 2.3	18.8–27.2	20.9 ± 1.6	18.3–24.4	0.006
HR_max_ (beats·min^−1^)	194.6 ± 6.3	176–207	195.5 ± 4.6	184–200	0.574
VO_2max_ (ml·kg·min^−1^)	53 ± 6.03	41–63	44.2 ± 5.6	32–54	<0.001
V_max_ (km·h^−1^)	17.8 ± 1.4	14–20	13.8 ± 0.5	13–15	<0.001
LA_max_ (mmol·L^−1^)	13 ± 2.2	9.7–18.6	11.1 ± 2.0	9–15	<0.001
Experience (y)	16.04 ± 4.1	10–30	13.2 ± 3.8	10–22	0.011
Table tennis training volume (h/week)	17.38 ± 2.92	12–22	17.92 ± 2.69	14–22	0.894
Complementary training (h/week)	2.79 ± 1.32	1–5	2.83 ± 1.34	1–5	0.975

BMI: body mass index; LA: lactate; V: velocity; HR: heart rate.

**Table 2:** Game temporal structure analysis.

Variable	Men		Women		
	Match				
	M ± SD	Range	M ± SD	Range	*p*
TT (s)	2256.05 ± 979.7	993.6–3507.7	1469.4 ± 544.2	783.1–2379.1	0.024
RPT (s)	395.06 ± 149.3	217.6–651.2	360.4 ± 131.4	194.08–693.6	0.553
TRT (s)	1860.9 ± 838.2	775.9–2896.4	1104.4 ± 459.1	589.1–1954.8	0.012
GD (s)	0.27 ± 0.05	0.20–0.38	0.41 ± 0.13	0.25–0.57	0.006
	Rally				
RPT (s)	3.6 ± 0.3	3.01–4.2	4.3 ± 1.07	3.08–6.4	0.035
TRT (s)	13.6 ± 2.7	9.1–19.3	11.2 ± 2.7	6.9–16.5	0.042

TT: total time; RPT: real playing time; TRT: total resting time; GD: game density.

**Table 3:** Physiological and metabolic analysis: cardiac and LA responses during the SC.

Variable	Men		Women		
	M ± SD	Range	M ± SD	Range	*p*
HR_max_ (beats·min^−1^)	163.9 ± 8.7	147–177	155.4 ± 8.01	139–167	<0.001
HR_min_ (beats·min^−1^)	111.3 ± 12.7	92–138	104.5 ± 7.5	93–118	0.029
HR_mean_ (beats·min^−1^)	138.7 ± 12.08	114–159	137.2 ± 6.03	127–148	0.572
HR_mean_ (%HRmax)	71.43 ± 7.08	57.29–82.39	70.25 ± 4.15	65.15–80.43	0.485
LA_basal_ (mmol·L^−1^)	1.01 ± 0.15	0.75–1.30	1.00 ± 1.14	0.73–1.24	0.780
LA_max_ (mmol·L^−1^)	1.8 ± 0.2	1.4–2.5	1.5 ± 0.2	1.1–1.9	<0.001

HR: heart rate; LA: lactate.

## Data Availability

The data presented in this study are available on request from the corresponding author. The data are not publicly available due to privacy.

## References

[1] Pradas F., Ara I., Toro V., Courel-Ibáñez J. (2021). Benefits of Regular Table Tennis Practice in Body Composition and Physical Fitness Compared to Physically Active Children Aged 10–11 Years. Int. J. Environ. Res. Public Health.

[2] Picabea J.M., Cámara J., Yanci J. (2021). Physical Fitness Profiling of National Category Table Tennis Players: Implication for Health and Performance. Int. J. Environ. Res. Public Health.

[3] de Mello Leite J.V., Barbieri F.A., Miyagi W., de Souza Malta E., Zagatto A.M. (2017). Influence of game evolution and the phase of competition on temporal game structure in high-level table tennis tournaments. J. Hum. Kinet..

[4] Kondrič M., Furjan-Mandić G., Kondrič L., Gabaglio A. (2010). Physiological demands and testing in table tennis. Int. J. Table Tennis Sci..

[5] Pradas F., Martínez P., Rapún M., Bataller V., Castellar C., Carrasco L. (2011). Assessment of table tennis temporary structure. Int. J. Table Tennis Sci..

[6] Michael K., Theofilos P., Aikaterini V. Real play time in table tennis matches in the XXVIII Olympic Games Athens 2004. Proceedings of the Book 10th Anniversary ITTF Sports Science Congress.

[7] Pradas de la Fuente F., Salvá Martínez P., González Campos G., González Jurado J.A. (2015). Analysis of performace indicators that define the modern table tennis. J. Sport Heal. Res..

[8] Zagatto A.M., Morel E.A., Gobatto C.A. (2010). Physiological responses and characteristics of table tennis matches determined in official tournaments. J. Strength Cond. Res..

[9] Zagatto A.M., Kondric M., Knechtle B., Nikolaidis P.T., Sperlich B. (2018). Energetic demand and physical conditioning of table tennis players. A study review. J. Sports Sci..

[10] Milioni F., Leite J.V.d.M., Beneke R., De Poli R.A.B., Papoti M., Zagatto A.M. (2018). Table tennis playing styles require specific energy systems demands. PLoS ONE.

[11] Kondrič M., Zagatto A.M., Sekulić D. (2013). The physiological demands of table tennis: A review. J. Sports Sci. Med..

[12] Zagatto A.M., de Mello Leite J.V., Papoti M., Beneke R. (2016). Energetics of Table Tennis and Table Tennis–Specific Exercise Testing. Int. J. Sports Physiol. Perform..

[13] Yuza N., Sasaoka K., Nishioka N., Matsui Y., Yamanaka N., Ogimura I., Takashima N., Miyashita M. (1992). Game analysis of table tennis in top Japanese players of different playing styles. Int. J. Table Tennis Sci..

[14] Sperlich B., Koehler K., Holmberg H.-C., Zinner C., Mester J. (2011). Table tennis: Cardiorespiratory and metabolic analysis of match and exercise in elite junior national players. Int. J. Sports Physiol. Perform..

[15] Castellar C., Pradas F., Carrasco L., De La Torre A., González-Jurado J.A. (2019). Analysis of reaction time and lateral displacements in national level table tennis players: Are they predictive of sport performance?. Int. J. Perform. Anal. Sport.

[16] Feliu J., Ventura J.L., Segura R., Rodas G., Riera J., Estruch A., Zamora A., Capdevila L. (1999). Differences between lactate concentration of samples from ear lobe and the finger tip. J. Physiol Biochem..

[17] Pradas F., Floría P., González-Jurado J.A., Carrasco L., Bataller V. (2012). Development of an observational tood for table tennis analysis. J. Sport Heal. Res..

[18] Altman D.G. (1991). Practical Statistics for Medical Research Chapman and Hall.

[19] Kasai J., Ohta A., Jung T.E., Mori T. (2010). Research on table tennis players cardio-respiratory endurance. Int. J. Table Tennis Sci..

[20] Katsikadelis M., Pilianidis T., Misichroni A. (2010). Comparison of Rally Time in XXIX Beijing (2008) and XXVIII Athens (2004) Olympic Table Tennis Tournaments. Int. J. Table Tennis Sci..

[21] Pradas F., Pinilla J.M., Quintas A., Castellar C. (2014). Game characteristics and time structure in high level table tennis. Rev. Int. Deport. Colect..

[22] Fernandez-Fernandez J., Jose G., Moya-Ramon M., Cabello-Manrique D., Mendez-Villanueva A. (2013). Gender differences in game responses during badminton match play. J. Strength Cond. Res..

[23] O’Donoghue P., Ingram B. (2001). A notational analysis of elite tennis strategy. J. Sports Sci..

[24] Torres-Luque G., Ramirez A., Cabello-Manrique D., Nikolaidis T.P., Alvero-Cruz J.R. (2015). Match analysis of elite players during paddle tennis competition. Int. J. Perform. Anal. Sport.

[25] Smekal G., von Duvillard S.P., Rihacek C., Pokan R., Hofmann P., Baron R., Tschan H., Bachl N. (2001). A physiological profile of tennis match play. Med. Sci. Sports Exerc..

[26] Cabello D., Padial P., Lees A., Rivas F. (2004). Temporal and Physiological Characteristics of Elite Women’s and Men’s Singles Badminton. Int. J. Appl. Sport. Sci..

[27] Chen H.L., Wu C.J. (2011). Physiological and Notational Comparison of New and Old Scoring Systems of Singles Matches in Men’s Badminton. Asian J. Phys. Educ. Recreat..

[28] Zagatto A.M., Papoti M., Gobatto C.A. (2008). Validity of critical frequency test for measuring table tennis aerobic endurance through specific protocol. J. Sports Sci. Med..

[29] Allen G.D. (1991). Physiological-characteristics of elite Australian table-tennis athletes and their responses to high-level competition. J. Hum. Mov. Stud..

[30] Suchomel A. (2010). A comparison of exercise intensity on different player levels in table tennis. Int. J. Table Tennis Sci..

[31] Baron R., Petschnig R., Bachl N., Raberger G., Smekal G., Kastner P. (1992). Catecholamine excretion and heart rate as factors of psychophysical stress in table tennis. Int. J. Sports Med..

[32] Djokic Z. (2004). Heart rate monitoring of table tennis players. Proceedings of the Science and Racket Sports III: The Proceedings of the Eighth International Table Tennis Federation Sports Science Congress and The Third World Congress of Science and Racket Sports.

[33] Barchukova G.V., Salakova E.V. (1991). Ergometric characteristics of table tennis. Sov. Sport. Rev. Dec.

[34] Hoshino S. (2013). Psychophysiological evaluation of cardiovascular response on the observational and practical task dificulty. Br. J. Sports Med..

[35] Iimoto Y., Yoshida K., Yuza N. (2002). Rebound characteristics of the new table tennis Ball; Differences between the 40 mm (2.7 g) and 38 mm (2.5 g) balls. Int. J. Table Tennis Sci..

[36] Tang H., Mizoguchi M., Toyoshima S. (2002). Speed and spin characteristics of the 40 mm table tennis ball. Int. J. Table Tennis Sci..

[37] Zhang H., Hohmann A., Lees A., Kahn J.-F., Maynard I.W. (2004). Table tennis after the introduction of the 40 mm ball and the 11 point format. Science and Racket Sports III.

[38] Jensen-Urstad M., Svedenhag J., Sahlin K. (1994). Effect of muscle mass on lactate formation during exercise in humans. Eur. J. Appl. Physiol. Occup. Physiol..

[39] Hafen P.S., Vehrs P.R. (2018). Sex-related differences in the maximal lactate steady state. Sports.

[40] Korhonen M.T., Suominen H., Mero A. (2005). Age and sex differences in blood lactate response to sprint running in elite master athletes. Can. J. Appl. Physiol..

[41] Martin C., Favier-Ambrosini B., Mousset K., Brault S., Zouhal H., Prioux J. (2015). Influence of playing style on the physiological responses of offensive players in table tennis. J. Sports Med. Phys. Fitness.

